# Germany and the Pan-American Medical Congress

**Published:** 1893-03

**Authors:** 


### Correspondence.

(*Pan-American Medical Congress Bulletin.*)

### GERMANY AND THE PAN-AMERICAN MEDICAL CONGRESS.

### AN OPEN LETTER FROM PROFESSOR CZERNY, OF HEIDELBERG, AND REPLY BY SECRETARY-GENERAL REED.

(Translated from *Deutsche Medicinische Wochenschrift*, January 12, 1893, p. 47.)

### An Open Letter

*To Claudius H. Mastin, M. D., LL. D., Mobile, Ala., President of the American Surgical Association, in reference to the Pan-American Medical Congress:*

Honored Sir and Colleague—You were kind enough to extend to me, December 3, 1892, a personal invitation to attend the Pan-American Medical Congress, to be held at Washington, September 5 to 8, 1893. According to the preliminary announcement, and to your communications, the Congress, in connection with the Columbian Exposition at Chicago, will offer so many attractions that I exceedingly regret not to be able to accept so amiable an invitation extended to me by one of the most prominent members of the body of American physicians.

As the reasons which prevent me from attending might be of interest to the German physicians, you will certainly pardon my desire to make these reasons more generally known in this manner.

At the first glance one might have been impressed with the idea that the Pan-American Congress was intended to be a rival to the long-prepared International Medical Congress, which is to be held at Rome from September 24 to October 1, 1893. However, as at the former international expositions, almost always contemporary medical and scientific congresses took place, it appears but just that the American physicians should also avail themselves of the opportunity of meeting on their own continent. But since the physicians of all civilized countries are united in the humane endeavor to rather mitigate than increase difficulties between nations and continents, where such exist, I therefore think any intention to injure the International Congress by the Pan-American, must be entirely excluded.

Perhaps the meeting of the International Congress might be postponed eight days, which, for several reasons, would be more desirable. With earnest intentions and favorable weather, it ought to be possible to make the trip, with the modern fast steamers, from Washington to Rome, from September 9th to September 23d. *It would not be a bad idea if those members who desire to make the journey, would do so jointly, directly from Washington to Rome, on a steamer chartered expressly for that purpose,* in order to bring the greetings of the rising capital of the promising West to the old Metropolis of European civilization.

A more serious consideration to visit the Pan-American Congress is entertained by me regarding the question of languages. In section ninth of your programme it says : "The languages of the Congress shall be Spanish, French, Portuguese, and English." The German language is probably excluded because it is nowhere official language in America. If this consideration should have prevailed, then the Dutch and Danish languages ought to have been permitted, since these languages are in official use in America. Be this as it may, *I do not think that the physicians of Germany can take part in the proceedings of the Pan-American Medical Congress, if they are not permitted to read their papers in German*, while any other language but the English is admitted at the Congress. It must be remembered that also at the International Congress "Remarks are permitted in any other language, if any of the members are willing to translate them into one of the four official languages."

I shall not mention the work done continually for the science of medicine in the German language, but I desire to refer to the great number of prominent American physicians who have received the best part of their education in German schools; and to the numerous German physicians who practise with success in America, and who have added so much to the great reputation in which American medical literature is at present held in the whole world. Indeed, I am inclined to believe that North and South American physicians will frequently be able to communicate with each other in the German language, learned by them in our universities. If I am not mistaken in this, I certainly think that the Executive Committee of the Pan-American Medical Congress should pass a resolution which would enable German physicians to visit the Congress, provided a participation on our part is at all desired.

I shall be exceedingly gratified if my suggestions should find favor on the other side of the ocean; and if I should be thus enabled, dear sir and colleague, to personally enter into friendly relations with you in Washington.

With best wishes and the compliments of the season, I am,

Yours most respectfully,

DR. CZERNY,

*Honorary Member of the American Surgical Association.*

HEIDELBERG, December 28, 1892.

(REPLY.)

THE PAN-AMERICAN MEDICAL CONGRESS.

*Office of the Secretary-General,*
311 Elm Street.
CINCINNATI, February 14, 1893.

*Professor V. Czerny, Heidelberg:*

MY DEAR DOCTOR—My distinguished colleague, Dr. C. H. Mastin, has referred to me for official reply your open letter addressed to him and published in the *Deutsche Medicinische Wochenschrift* for January 12th of this year.

A careful reading of your valued communication leads me to the conclusion that you, in common with other distinguished German *savants*, hesitate in accepting an invitation to attend the Pan-American Medical Congress, (1) because the German profession is not officially invited by the Executive Committee to become a constituent part of the Congress; (2) because the German language is not one of the official languages of the Congress, and (3) because a general participation on the part of yourself and *confrères* might be construed into disloyalty to the International Medical Congress which is to meet in Rome in the same month.

In reply I beg to state that the occasion for holding a medical Congress in the United States in 1893, is the fact that a large number of physicians will be in this country in attendance upon the World's Columbian Exposition. This attendance will be largely, although not by any means exclusively, from the countries of the Western Hemisphere. It would have been very desirable, indeed, to have arranged an organization which would have embraced all the countries of the world. The medical profession of the United States, however, acknowledges allegiance to the World's International Congress which is to meet in Rome. To have attempted an organization in Germany, or any other European countries, in the interest of the American meeting, would have been in violation of our loyalty to the International Congress, while an official invitation to the government and medical societies of Germany and other European countries to send delegates to the Washington meeting would have been almost equally inimical to the interests of the Rome Congress. It was, therefore, resolved that the organization should be limited to the American countries, and that while it was desirable to secure the attendance of our distinguished *confrères* from Europe as guests, invitations to that end should be strictly personal in character, and should be issued by the general officers and presidents of sections, at their discretion.

The languages chiefly spoken by the peoples of the various constituent countries of the Congress are Spanish, Portuguese, and English, and these were accordingly selected by the Committee as the official languages of the Congress. French, which is the language of important colonies and communities, was subsequently added, at the instance of our *confrères* in Brazil, who employ it largely in scientific communication, as, indeed, do a large proportion of the physicians of both the English and Spanish-speaking countries. Danish and Dutch are not included simply for the reason that it is extremely, indeed practically, impossible to deal with them satisfactorily in a literary way in this country. It was hoped that delegates from countries and colonies speaking other than Spanish, Portuguese, English, and French would furnish their remarks on papers in one of the official languages. This was so thoroughly understood by the Committee, and has become such a well-established usage at international congresses, that it was not deemed necessary to state it explicitly; but I shall communicate the suggestion which you kindly make to the Executive Committee, when, I have no doubt, it will be made definite in the By-laws.

As early as December, 1891, I opened correspondence by telegraph with the President of the eleventh International Congress, and subsequently with Professor Maragliano, of Genoa, the Secretary-General, asking that the date of the Rome meeting be arranged so as to permit us to send delegates from Washington. The date of the International Congress was accordingly changed from the 17th to the 24th of September, which will give us sixteen days in which to go from Washington to Rome. Arrangements were begun in February of last year for a special sailing of a steamer September 9th, direct to Italy, by way of the Azores and Gibraltar, to take those desiring to attend the Rome meeting, a special reduced rate being accorded for the occasion. My present correspondence indicates that a large number will avail themselves of this privilege. It is highly gratifying to note that the expediency of this plan has occurred quite independently to one so conversant with affairs as yourself.

Permit me to say, in conclusion, that our European *confrères* who may honor the Pan-American Medical Congress with their presence will be accorded every linguistic privilege, that arrangements have already been made for their return to Italy in time for the International Congress, and that in the event of their coming they will be greeted with a most cordial American welcome.

Very sincerely yours,

CHARLES A. L. REED,

*Approved:* *Secretary-General P.-A. M. C.*

William Pepper, *President.*

Claudius H. Mastin, *Member of the Board of Trustees.*

---

Unfortunately, the remarks of Dr. M. A. Crockett, in closing the discussion on his part on the group of papers read before the Medical Society of the County of Erie, were left out of their proper place. They are printed below, instead of on page 479, where they belong. Dr. Mann's remarks should precede Dr. Crockett's, otherwise the discussion is in its proper order.

Dr. Crockett in closing: Several of the speakers are apparently in favor of making cultures of the gonococcus. Their method is to use the wife as the culture medium; if she escapes the disease the husband was cured. This is not the plan I suggested in my paper. No one maintains that *every* case of gonorrhea results in a pus tube; as well say exposure to small-pox means to contract the disease. You *must* admit that gonorrhea *may* produce certain disastrous results. This is enough to make it imperative to assure ourselves as to the question of cure before marriage is allowed. A case is cured when no more gonococci can be found by means of culture tests. It is perfectly feasible for a pathologist to make this test, and certainly the question is serious enough to warrant a little trouble.

Dr. Frogie's methods, as quoted by Dr. Pryor, are tolerably reliable, but much less so than culture tests, and certainly quite as much trouble. I agree with Dr. Pryor that in certain cases we should find the gonococcus before stating the disease to be gonorrheal. On the other hand, there are certain clinical symptoms to gonorrhea, just as there are to phthisis, and we can be reasonably certain in many cases without looking for the bacteria to confirm us.

